# An evaluation of modifications to NHS Health Checks: A study protocol

**DOI:** 10.1371/journal.pone.0330368

**Published:** 2025-09-22

**Authors:** Nigel Lloyd, Charis Bontoft, Katherine Barrett, John Jackson, Julia Jones, Lisa Miners, Nigel Smeeton, Adam P. Wagner, Amander Wellings, Katherine Brown

**Affiliations:** 1 University of Hertfordshire, Department of Psychology, Sport and Geography, School of School of Health, Medicine & Life Sciences, Hatfield, United Kingdom; 2 University of Hertfordshire, Centre for Research in Public Health and Community Care, (CRIPACC), School of Health, Medicine & Life Sciences, Hatfield, United Kingdom; 3 University of East Anglia, Norwich Medical School, Norwich, United Kingdom; 4 NIHR Applied Research Collaboration (ARC) East of England (EoE), Douglas House, United Kingdom; Khyber Medical University, PAKISTAN

## Abstract

Cardiovascular disease (CVD) is the leading cause of death globally; in the UK it contributes to a quarter of all deaths. In 2009, the UK National Health Service launched the NHS Health Check (NHSHC) programme to address CVD by assessing all adults aged between 40 and 74 years for CVD risk factors. Encouraging uptake of NHSHCs has proved challenging and areas for development have been identified nationally, prompting modifications to NHSHC practice in some localities. This protocol article describes a programme of research that will evaluate the impact of NHSHC modifications on attendance and outcomes in a large English local authority area. Modifications to NHSHC delivery (delivery of a modified NHSHC by a healthy lifestyle service rather than via general practice led delivery) and NHSHC invitation processes (implementation of text message prompts and reminders and an additional online booking option) will be evaluated. The research consists of six workstreams within a mixed methods framework: 1) Quantitative analysis of client NHSHCs records; 2) Focus groups with healthy lifestyle staff involved in coordinating and delivering the modified NHSHCs sessions; 3) Interviews with staff from pilot GP practices whose roles involve the coordination or delivery of NHSHCs; 4) Interviews with clients who have attended a healthy lifestyle service NHSHC; 5) Health economic resource and cost evaluation; 6) Data analysis, synthesis, and dissemination. The research programme’s breadth and its novel nature, mean that it will provide valuable findings for those commissioning and delivering NHSHCs nationally, and for the wider public health community.

## Introduction

### Cardiovascular disease: background and growing prevalence

Cardiovascular disease (CVD) is a general term for conditions affecting the heart or blood vessels such as coronary heart disease, heart failure, and stroke. It is the leading cause of death globally, accounting for an estimated 17.9 million deaths in 2019, 32% of global deaths [1]. In the UK, approximately seven million people are living with CVD, and it contributes to a quarter of UK deaths [2]. Economic costs of CVD are significant, with CVD-related health and social care costs in England estimated to be £12 billion annually, and societal costs £28 billion annually [3]. CVD is a leading cause of health inequalities [4] and the largest cause of premature mortality in deprived areas of England [5].

The incidence of CVD increases rapidly with age, is higher for males than females, and has a positive association with deprivation, the impact being greatest in the most socio-economically disadvantaged groups [6]. Cardiovascular disease (CVD) risk has also been shown to vary across ethnic groups. In the UK, South Asian individuals have been found to have an increased risk of CVD overall [7], but for specific cardiovascular diseases, reported associations with ethnicity are mixed. For instance, in a study of White, Black and South Asian patients, George et al. [8] found that, relative to the White group, there was a raised risk of angina for those in the South Asian group but a decreased risk for Black individuals. Risk of heart failure was similar for all three ethnic groups whereas stroke risk was relatively higher for individuals of both South Asian and Black ethnicities. There is a positive causal link between smoking and CVD [9].

### The NHS Health Check

The NHS Health Check (NHSHC) programme was launched by the UK National Health Service (NHS) in 2009 and is a cornerstone of CVD prevention in the UK. The programme aims to assess older adults for CVD risk factors by providing people aged between 40 and 74 years, without pre-existing health conditions, with a health check free at point of access. Eligible individuals are invited to an NHSHC by their GP or local council every five years, or can self-refer [10]. Throughout this document the abbreviations NHSHC and HC will be used interchangeably to refer to the NHS Health Check.

NHSHCs are conducted by a healthcare professional, typically a healthcare assistant (HCA) or nurse, although they may also be delivered by a doctor or pharmacist. They involve the health professional taking measurements such as height, weight, waist size, blood pressure, and cholesterol. Attendees are also asked to provide details of family history of medical conditions, alcohol and tobacco consumption, and physical activity level. Results are used to calculate a CVD risk score, known as a QRISK score [11], and attendees also receive an assessment of their body mass index (BMI) and diabetes risk [10]. At the end of the NHSHC, attendees receive feedback on their health status and, where relevant, advice, guidance, and support on beneficial lifestyle changes and appropriate clinical and behavioural approaches to reduce the risk of CVD and improve overall health and wellbeing. This may include signposting or referral to a General Practitioner (GP) to consider pharmacological interventions or to ‘lifestyle’ services (such as a weight management, smoking cessation, or addiction services) [10].

Research suggests that NHSHCs may positively impact health outcomes, particularly through the identification of CVD risk. For example, Usher-Smith et al. [12] conducted a rapid synthesis of the published evidence on NHSHCs and highlighted small increases in disease detection and greater statin prescription for attendees, with a new case of raised blood pressure found in three to four health checks and a person with cardiovascular disease risk (>20%) identified in six to ten. Changes in blood pressure, cholesterol and BMI were also significantly larger in NHSHC attendees than matched non-attendees. Similarly, Tanner et al.’s, 2022 review found that NHSHCs lead to increased detection of CVD risk factors [13]. Research has also identified increased referral to health improvement services following NHSHCs [14].

However, the effect of the NHSHC on attendees’ lifestyle and health-related behaviours is unclear, with limited research examining post NHSHC behaviour change and a need for additional research in this area [13].

### Encouraging uptake of NHSHCs

The NHSHC programme costs hundreds of millions of pounds per year to deliver, with the most recent cost estimate approximately £450 million annually [15]. However, encouraging uptake of health checks has proved challenging, with inequity identified in the likelihood of different groups attending. Research suggests that fewer than half of those eligible attend a health check [16–18] and uptake has been found to be higher for older people and females, and lower for those living in deprived areas [16,17,19]. Ogunlayi et al.’s recent study found that individuals from some minority ethnic backgrounds and those living in the most deprived localities were less likely to be invited to a health check or take up the invitation to attend [20].

A 2018 systematic review to understand barriers to NHSHC attendance identified lack of awareness of the availability of NHSHCs, misunderstanding of the purpose and benefits of the health check, time and access constraints, and privacy and confidentiality concerns, among the main barriers [21]. Studies have highlighted significant variation in NHSHC invitation and delivery models and practices, and those delivering health checks have often modified practice to improve uptake or delivery [12,22,23].

The mode and content of NHSHC communications have been studied extensively and found to influence the likelihood of health check attendance. For instance, telephone and verbal invitations appear to be more effective than postal invitations [24,25], and face-to-face invitations more effective than letter or telephone [19]. Additionally, the positive impact on health check attendance, of amending invitation letters or using text messaging, is well-established [26,27]. Specifically, studies have shown that the precise framing and wording of health check invitation letters [28] and the use of text message primers or reminders [29] can positively impact NHSHC uptake.

However, the relationship between communication methods and NHSHC attendance appears far from straightforward, with studies suggesting that the effectiveness of invitation methods may vary depending on invitee characteristics [19,26]. Cook et al.’s 2016 study, for example, found that ethnicity and gender appeared to play a key role in determining invitee responses to different forms of invitation communication. They cite misunderstandings about the NHS system, language barriers, and a lack of trust as potential factors influencing the effectiveness of communication methods and lower NHSHC uptake among immigrant communities — specifically Polish migrants in this study [19]. Cook et al. are among those who suggest that tailored approaches to communicating with different population groups about health checks may be necessary to address disparities in uptake [19,30].

Beyond the invitation process itself, studies suggest that the setting in which the NHSHC is delivered may positively influence uptake, as checks undertaken in community venues such as pharmacies, retail spaces or places of worship may be more accessible or acceptable to patients than those delivered in GP practices [30–32]. It is also reasonable to expect that the practitioner or organisation delivering the NHSHC could affect health check experiences or outcomes. Existing evidence suggests that the role of the medical professional conducting the NHSHC — whether a doctor, healthcare assistant, or pharmacist — may influence outcomes such as medication prescribing and referrals to lifestyle services [33], and that delivery by trained non-clinical staff, such as healthy lifestyle advisors, is feasible and may be experienced positively by patients [34]. Research also highlights the importance of appropriate training for those delivering the health check [31]. However, there is limited research exploring the impact of *who* delivers the health check and no studies to date have examined differences in patient experience or outcomes between NHSHCs delivered by clinical GP practice staff, such as nurses and HCAs, and non-clinical staff.

Recognising scope for improvement of the NHSHC programme, local authorities have begun to adapt the health check intervention to better address the development of diseases and long-term conditions in their localities.

### The current study

This study will evaluate the modifications made to NHSHC invitation and delivery in a large local authority area in the East Midlands region of England. The local authority has identified ‘working to support people to live healthy lives’ as a strategic priority and funds a healthy lifestyles service delivered by specialist healthy lifestyles practitioners. The service – which includes support and referrals for smoking cessation, weight management, and physical activity – is available to anyone within the locality.

In June 2022, the local authority began a pilot project in which the delivery of NHSHC was modified in ten GP practice areas. A key modification involved staff from the healthy lifestyle service, rather than GP practice staff, delivering NHSHCs (in some pilot practice areas, some delivery of NHSHCs by GP practice staff continues alongside healthy lifestyle service health check delivery). In the pilot practice areas, eligible residents who are registered with the pilot GP practice either have the option of choosing to attend a ‘GP-led’ or ‘lifestyle service’ health check, or will receive a lifestyle service health check as standard (depending on the practice). There are several differences between a GP-led health check and a lifestyle service health check:

- A lifestyle service health check lasts for approximately 45–60 minutes, compared to the shorter GP-led health check. While the length of NHSHCs is not mandated by the NHS, NHS publicity suggests they typically last about 20–30 minutes [10] and in the study area, GPs were funded to complete an NHSHC within 30 minutes. However, although some individual GP practices record the duration of their health checks, the actual time allocated to GP-led NHSHCs is not collated at the study area level, and reliable data on their duration are therefore unavailable. Nevertheless, several published studies have identified significant variation in NHSHC duration, with some checks lasting as little as five to seven minutes [22,35].
- The additional time in a lifestyle service health check is intended to allow for further lifestyle conversations, referrals, and signposting.
- Staff delivering a lifestyle service health check are holistic lifestyle experts who have specific referral expertise and can signpost to a wide variety of wellbeing services. A standard GP-led health check may be delivered by a nurse, doctor, pharmacist, or healthcare assistant and may be just one of many clinical responsibilities.
- As standard, the lifestyle service health check offers an HbA1c test to everyone — a blood test that measures average blood glucose levels and consequently the presence or development of diabetes. In the study area, there is variation between GP practices in whether an HbA1c test is offered as part of the GP-led health NHSHC. For example, some offer an HbA1c test as standard (either prior to or during the NHSHC), while others only offer it if clinical risk symptoms are identified.
- A lifestyle service health check incorporates all GP-led checks and measures but additionally includes a ‘health and wellbeing MOT’. The MOT adopts a holistic wellbeing approach, focussing on broader domains than a standard NHSHC, including assessment of emotional wellbeing, financial and debt issues, and employability concerns. Depending on the client’s responses, they may be referred to receive additional support from the lifestyle service, NHS services, or other third-sector providers.

In addition to lifestyle service health check delivery, the local authority has made two further modifications to NHSHCs in the ten pilot areas. These will also be the focus of this evaluation. Firstly, beginning in June 2023, postal reminders sent to those eligible for an NHSHC to remind them to book an NHSHC appointment were supplemented by text message prompts and reminders. An initial text message is sent prior to an NHSHC invitation letter and a subsequent text is sent to remind the client to book an appointment. Secondly, from October 2023, those eligible for health checks have had the additional option of booking their NHSHC appointment online. The previous telephone booking option remains in place. Our evaluation will therefore focus on three NHSHC modifications:

1. Modifications to NHSHC delivery – delivery is a healthy lifestyle service rather than GP-led.
2. Modification to NHSHC invitation process – implementation of text message prompts and reminders.
3. Modification to NHSHC invitation process – implementation of an online booking option.

**Fig 1:**
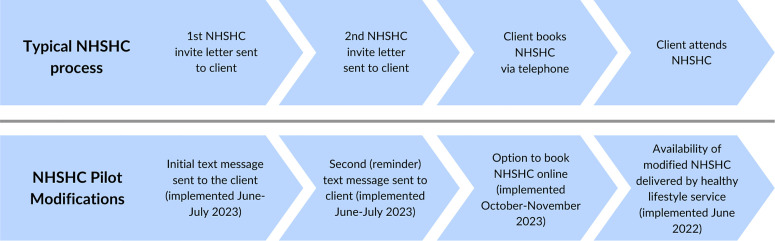
A typical NHSHC process and the modifications made to the invitation and delivery process as part of the current pilot.

Fig 1 illustrates the standard NHSHC process, along with the modifications introduced to the invitation and delivery procedures as part of the current pilot.

### Study aims and research questions

The study aims firstly, to investigate the impact of modifications to NHSHC invitation and session delivery on health check attendance, outcomes, and resources. Secondly, to explore staff and client experiences of the modifications made to health check practice.

To address the impact of modifications to the NHSHC invite process, the study will investigate the impact of text message prompts and reminders, and the online booking option, on the uptake of the NHSHCs. We will also investigate whether the lifestyle service health checks lead to any changes in health check outcomes (e.g., numbers of referrals or new prescriptions), and outcomes data will be contrasted between the standard GP-led and lifestyle service led NHSHCs. In addition, staff and client experiences of the lifestyle service health check will be explored, as will changes to clients’ health-related knowledge and planned behaviour.

We aim to answer the following research questions:

1. Does the implementation of a) text message prompts and reminders, and b) an NHSHC online booking option, lead to changes in the proportion of those invited attending an NHSHC?
2. Does delivery of modified NHSHCs by the healthy lifestyles service, which includes Health and Wellbeing MOTs, lead to any changes in NHSHC outcomes?
3. What are staff experiences of the modified NHSHC?
4. What are clients’ experiences of the modified NHSHC and how do clients feel it has impacted their current and future health and wellbeing?
5. What are the costs and resources required for the modified NHSHC delivery and how do they compare to costs and resources for standard GP-led NHSHC delivery?

## Materials and methods

### Ethical considerations and declarations

Ethical approval has been received through the University of Hertfordshire Health, Science, Engineering and Technology Ethics Committee with Delegated Authority (protocol number: LMS/SF/UH/05540). The NHS Health Research Authority (HRA) decision tool has been consulted and has confirmed that NHS Research Ethics Committee review and approval is not required as the study is ‘service evaluation’ rather than ‘research’.

### Patient and public involvement (PPI)

A PPI group, known as the Public Involvement in Research group (PIRg), is an integral part of the research team conducting the current evaluation. Two ‘embedded’ PIRg members will work alongside the research team as co-researchers for the duration of the evaluation. The embedded PIRg members will attend project meetings, review project materials, and contribute to the production of project outputs. Their input will be complemented by public contributors from the pilot area locality, who will be recruited with the assistance of local voluntary and community sector organisations. These public contributors, who will meet NHSHC eligibility criteria, will provide additional local public perspective on the evaluation. They will review public-facing project materials and attend project Advisory Group (AG) meetings as full AG members. Embedded PIRg members and local public contributors will be remunerated for their time in line with NIHR guidance [36].

### Study design overview

The study will adopt a mixed methods design, incorporating analysis of routinely collected quantitative data, alongside qualitative data collection and analysis. A convergent, parallel mixed methods approach will be utilised [37], applying both methods concurrently, and bringing them together at the interpretation stage. The mixed method approach will allow for complementarity in the analysis process [38].

We will use the six APEASE criteria [39] as a framework for evaluating the NHSHC modifications. The criteria, summarised in Table 1, can be utilised for a range of evaluation purposes including for formal evaluation of existing interventions [39] and offer a thorough and methodical framework for evaluating health interventions [40]. They will be used here as a framework through which to interpret, understand and report our findings; the criteria encompass the key components and concepts unpinning our research aims, and all our data collection and analysis will inform one or more of them.

**Table 1:** Summary of APEASE criteria (adapted from West et al., 2019 [39]).

Criterion	Description
Acceptability	How far is the intervention considered acceptable to key stakeholders? E.g., target groups, those involved in coordinating and delivering the intervention, community members and funders.
Practicability	Can the intervention be implemented as designed within the intended context, material and human resources? What are the practical considerations to its implementation?
Effectiveness	How effective is the intervention in achieving the desired objectives and what is the extent of its effectiveness?
Affordability	How affordable is the intervention when delivered at the scale intended? Is the necessary budget available to deliver the intervention? Does the intervention provide a good return on investment?
Side-effects	Does the intervention lead to any unintended adverse or beneficial outcomes?
Equity	How far does the intervention increase or decrease differences between advantaged and disadvantaged sectors of society?

Qualitative study results will be reported in accordance with the Consolidated Criteria for Reporting Qualitative Research [41].

The project has been divided into six distinct workstreams (WSs). Each seeks to answer specific research questions (RQs) or address RQs in a different way to provide for a well-rounded and robust evaluation. Table 2 provides a summary of each workstream and the study research question/s that it informs.

**Table 2:** Study workstreams (WSs) mapped to research questions (RQs).

WS	Summary	RQ1	RQ2	RQ3	RQ4	RQ5
1	Quantitative analysis of client NHSHCs records	✓	✓			
2	Focus groups with healthy lifestyle service staff involved in coordinating and delivering them modified NHSHCs sessions			✓		✓
3	Individual or joint interviews with staff from pilot GP practices whose roles involve the coordination or delivery of NHSHCs			✓		✓
4	Interviews with clients who have attended a healthy lifestyle service NHSHC		✓		✓	
5	Health economic resource and cost evaluation					✓
6	Data analysis, synthesis, and dissemination	✓	✓	✓	✓	✓

### WS1: Quantitative analysis of client NHSHC records

#### Aim.

To investigate whether delivery of lifestyle service NHSHCs, implementation of text message prompts and reminders, and introduction of a HC online booking option led to changes in:

a) attendance at NHSHCs based on those invited for a health check
b) NHSHC-related outcomes based on those who received a health check

#### Design.

As Fig 1 shows, from June 2023, postal invitations to attend an NHSHC were supplemented by text message prompts and reminders. In October of that year, online booking of NHSHCs was also made available. The relationships under investigation in this workstream are:

- the proportion of those invited for an NHSHC who attended a health check by the methods of communication in use;
- the proportion of NHSHC visits that led to specific health check related outcomes by type of health check delivery.

NHSHC data are managed, on behalf of the local authority, by an independent organisation. We are currently awaiting confirmation from the organisation, of the precise nature and format of data available. However, data on age group, gender, Index of Multiple Deprivation quintile, ethnicity, and smoking status – all of which have been associated with CVD risk – should be made available, with the categories for these variables yet to be decided. The precise statistical methods and the variables chosen for the analysis are therefore yet to be confirmed and will be dependent on the sample size and the data that can be provided at the level of the individual. However, given the planned timeframe of this study, records for around 10,000 individuals are expected to be available, indicating that the large-sample methods described below should be appropriate. If sample sizes are much lower than anticipated, the equivalent methods adapted for small samples will be considered.

#### Recruitment.

The study timeframe is the two years from start of June 2022 to the end of May 2024, which has been divided into:

- Period 1 (between June 2022 and May 2023) – both GP and healthy lifestyle service NHSHC delivery available but no additional methods of communication.
- Period 2 (between June 2023 and November 2023) – both GP and health lifestyle service NHSHC delivery available alongside text message prompts/reminders.
- Period 3 (December 2023 and May 2024) – both GP and health lifestyle service NHSHC delivery available alongside text message prompts/reminders and online NHSHC booking.

To assess uptake and attendance of NHSHCs, individuals will be followed up for the two months following the date of first invitation, so only individuals invited before the end of March 2024 will be analysed. Records selected will include those for May 2022, as individuals attending for a HC at the start of Period 1 should have been sent an invitation during that month.

Fully deidentified NHSHC data for individuals from practices involved with the pilot at any point will be obtained from the independent company managing the NHSHC data. Individual client records contain demographic information, observations recorded during the health check, and records of subsequent referrals. In addition, records show whether the individual received GP or health lifestyle service NHSHC delivery.

#### Analysis.

The unit of analysis will be the individual. Data will be analysed by time interval: Period 1 (no texts and no online booking); Period 2 (texts but no online booking); Period 3 (texts and online booking) and by type of delivery (GP; health lifestyle service delivery).

Individuals sent an NHSHC invitation will be grouped according to their attendance or nonattendance at a HC. Similarly, health check related outcomes will be analysed in terms of presence or absence of that outcome in the post-HC database records for that individual. For HC attendance, the proportion of those invited who attended a HC will be compared by Period using the chi-squared test, with individuals receiving GP delivery and health lifestyle service delivery combined. For each health check related outcome, the proportion of individuals who by attending a HC received the outcome appropriately will be compared by type of delivery (GP v. health lifestyle service), with individuals in the three Periods combined.

GP and health lifestyle service delivery will be compared stratified by Period, using loglinear modelling, to allow for period effects. To adjust for potential explanatory factors (e.g., age, gender), outcomes will be analysed using multivariable logistic regression, which models the logarithm of the odds of an event, such as a particular type of referral, occurring [42].

Not all practices remained in the pilot study throughout; additional sensitivity analyses will be performed based only on practices with complete participation. Stata Version 15.1 [43] will be used for the analyses.

#### Sample size.

Sample size will be determined by the number of records available for the practices involved in the study. As an indication, around 4,900 health checks are delivered per year. Therefore, there will be approximately 4,900 records for Period 1, 2,450 records for Period 2, and 1,640 records for Period 3.

### WS2: Focus groups with healthy lifestyle service health check staff involved in coordinating and delivering the modified health check sessions

#### Participants and recruitment.

Participants will be healthy lifestyle service staff currently involved in the coordination or delivery of the NHSHC pilot within GP practices. All relevant staff will be invited to participate (currently six in total). An email from the research team, containing a link to a secure online system, REDCap [44], will be circulated to all prospective participants. The link will allow participants to read a study participant information sheet and provide e-consent and basic details about themselves (e.g., job role, gender, and basic demographic details). Inclusion criteria:

- Be aged 18 years or older.
- Have capacity to consent to participate.
- Have provided informed consent to participate.
- At the point of registration to participate, be a healthy lifestyle service staff member involved in coordinating or delivering the modified HC sessions.

Recruitment will begin and complete during February 2024

#### Data collection.

Data will be collected via online focus groups using video conferencing software. Focus groups will be facilitated and moderated by two members of the research team and are expected to last no longer than one hour. Focus groups will be audio and video recorded, with consent for recording gained from participants prior to recording commencing. Audio recordings will be fully transcribed prior to analysis.

A focus group topic guide has been co-developed with the PPI co-researchers embedded as part of the research team. Topics explored during focus groups will include: aims and objectives of the lifestyle service NHSHC; participants’ and other stakeholders’ views on the lifestyle service NHSHCs (e.g., acceptability); the process of setting up and delivering lifestyle service NHSHCs within GP practices; content of the modified health check and structure of the health check session; barriers to health check engagement and outcomes and any differences between client groups; factors that promote/enable health check engagement and outcomes; challenges to effective delivery of the lifestyle service NHSHCs.

Focus groups will be conducted in late February 2024

#### Analysis.

Further detail on our approach to management and analysis of qualitative data can be found in the ‘WS6: Data analysis, synthesis, and dissemination’ section.

### WS3: Individual or joint interviews with staff from pilot GP practices whose roles involve the coordination or delivery of NHSHCs

#### Participants and recruitment.

Participants will be staff from the ten pilot GP practices whose roles involve the coordination or delivery of GP-led NHSHCs in their practices or supporting delivery of healthy lifestyle service NHSHCs. Participants will be recruited with the assistance of local authority partners involved in coordination of the pilot.

To explore experiences of GP practice staff and the impact of healthy lifestyle service delivery on their roles and workload, practice staff will be invited to attend an individual or joint interview (two interviewees) with a member of the research team. Joint interviews will involve staff members from the same GP practice.

Practices will be provided with information about the evaluation to circulate to these staff members; the information will include a link to a secure online system (REDCap) where potential participants will be able to read a participant information sheet and provide e-consent and contact details. Once consent has been gained, participants will be contacted by email or telephone to arrange a convenient interview time and date.

To ensure a range of GP practices are represented, we will aim to conduct interviews with staff members from at least four different practices. Other inclusion criteria are:

- Aged 18 years or older.
- Have capacity to consent to participate.
- Have provided informed consent to participate.
- At the point of registration to participate, be a healthcare professional working within one of the pilot GP practices whose role involves the coordination or delivery of NHSHCs.

Recruitment will begin in February 2024 and finish in April 2024

#### Data collection.

A maximum of ten, individual or joint, semi-structured, in-depth interviews will be conducted. Interviews will be no longer than one hour in duration, and it is anticipated that data will be collected remotely (i.e., using telephone or video conferencing software).

However, data may be collected via face-to-face interviews where this is preferable to the participant. A single research team interviewer will conduct each interview.

An interview topic guide will be produced ahead of interviews, which will be co-produced in collaboration with embedded PIRg members. Depending on staff member role, topics explored during interviews will include: extent of healthy lifestyle service NHSHC provision within the practice; the process of setting up and delivering a standard NHSHC; extent of joint working/liaison with the healthy lifestyle service; impact of the healthy lifestyle service NHSHC delivery on participant’s role and workload (including benefits and downsides); suggestions for improvement of NHSHC delivery.

Data collection will take place from March 2024 to May 2024

#### Analysis.

Further detail on our approach to management and analysis of qualitative data can be found in the ‘WS6: Data analysis, synthesis, and dissemination’ section.

### WS4: Interviews with clients who have attended a healthy lifestyle service NHSHC

#### Participants and recruitment.

To explore client experiences of the modified, healthy lifestyle service health check and motivators, barriers and enablers to engagement, we will recruit 10–12 participants who have recently (within the previous six weeks) received such an NHSHC.

The inclusion criteria are:

- Aged 18 years or older.
- Have capacity to consent to participate.
- Have provided informed consent to participate.
- Have received a modified, healthy lifestyle service NHSHC within the previous six weeks.

Following delivery of a modified NHSHC, healthy lifestyle service staff will hold a brief discussion with clients about the evaluation. They will provide information about the evaluation and what participation would involve. Where the client is interested in the possibility of participating, the staff member will present a secure REDCap site, via laptop or tablet, where they can register their interest in the study or e-consent to participate. An information sheet with details of the evaluation, the research team’s contact details, and a scannable QR code that links to the REDCap site will be provided to those potential participants who prefer not to register or e-consent immediately.

Once a pool of potential participants is generated, maximum variation sampling will be used to obtain a sample which provides variation in terms of gender, age, and ethnic background. Selected participants will be contacted by a member of the research team to arrange an interview (if consent has been given) or gain verbal consent or e-consent to participate. Where consent is provided verbally, a recording of consent will be made and kept on a secure drive. Interviews will be conducted as soon as possible after recruitment to minimise the time between the health check and the research interview, and thus maximise the likelihood of participants having an accurate recall of their heath check experience.

A ‘thank you’ shopping voucher of £20 will be offered to all of those who participate in an interview.

Recruitment will begin in February 2024 and finish in April 2024

#### Data collection.

A maximum of 12 individual, semi-structured, in-depth interviews with a selection of clients who have received a healthy lifestyle service health check.

We anticipate that most interviews will be conducted remotely (i.e., using telephone or Zoom). However, data may be collected via face-to-face interviews where this is preferable to the participant. Interviews will take no longer than one hour and will be conducted by a single research team member.

The interview topic guide will be co-produced in collaboration with embedded PIRg members. Topics explored during interviews will include: clients’ health check expectations and experiences; barriers to and motivations for health check attendance; the difference the health check has made to the client and any well-being related knowledge gained; and anticipated future wellbeing-related changes and motivations for these.

Data collection will take place from February 2024 to May 2024

#### Data analysis.

Further detail on our approach to management and analysis of qualitative data can be found in the ‘WS6: Data analysis, synthesis, and dissemination’ section.

### WS5: Health economic resource and cost evaluation

#### Aim.

To estimate the resources used (staff time and consumables) and associated cost per patient for attendance to either a modified or standard NHSHC health check (as delivered by a large local authority in the East Midlands region of England).

#### Design and data collection.

For this resource and cost evaluation, we will map the steps involved in delivering the modified and standard NHSHC. Relevant healthcare professionals and managers will be invited to an information gathering discussion in which we will discuss the different pathway delivery types, resources and the budgets associated with the different health check types.

The average cost per patient for each pathway will be estimated using a micro-costing approach. The primary cost perspective of the analysis will be that of the local authority funder. Where (health economic) resources (e.g., staff time) need to be costed, we will draw on costs from the appropriate programme and standard sources (e.g., Unit Costs of Health and Social Care 2022 Manual [45]) as needed, using the latest cost year for which data are available at time of analysis. Consumable costs will be sourced from the local authority. We will also seek the costs of implementing the online booking system and text message reminders.

#### Mapping and resource use of NHSHC pathways.

Summary diagrams, in Microsoft Excel, will be created listing the steps and resources used (staff time and consumables) for different NHSHC deliveries. From this we will estimate the associated costs required to deliver the different health checks (with separate costing scenarios for components identified as likely to vary – such as duration of standard check appointments and the staff type delivering them).

### WS6: Data analysis, synthesis, and dissemination

All interview and focus group data will be transcribed verbatim and uploaded into NVivo 14 software (QSR International) for coding and analysis.

Following transcription, all qualitative data will be analysed using Framework Analysis [46]. This approach offers a structured and systematic approach to managing qualitative data analysis across multiple researchers [47], which will help facilitate the involvement of PIRg members in the analytic process. We will use the following staged Framework Analysis process: familiarization with the data; coding; development of an analytical framework; applying the analytical framework, charting, and mapping and interpretation [47–49].

We will conduct both inductive and deductive coding to enable us to understand findings in terms of the APEASE criteria, while also allowing for exploration of staff and client experiences and providing scope for the identification of unanticipated themes relevant to the research questions. Our initial coding will utilise pre-selected codes related to the APEASE criteria while simultaneously allowing for inductive coding of transcripts. After Framework Analysis ‘charting’ has been conducted, themes will be identified under each APEASE criterion, with space allowed for identification of themes that lie outside the scope of APEASE but are relevant to our research questions.

Qualitative and quantitative data will be integrated at the ‘interpretation and reporting’ stage [50]. Qualitative research data collected during Workstreams 2, 3, and 4, and quantitative data from Workstreams 1 and 5, will be separately analysed as standalone workstreams before being brought together [51]. A mixed contiguous/weaving approach will be used for qualitative and quantitative data integration [52], allowing the research team to integrate findings from the quantitative outcomes and health economic analyses with qualitative analysis of staff and clients’ interview and focus group data. During the interpretation and reporting stage, the APEASE criteria will act as a framework through which we will interpret and report findings.

Our dissemination and knowledge mobilisation work will involve close collaboration with a broad range of stakeholders including: local authority representatives; public health practitioners; NHSHC specialists; policy experts; service users; and academics. This will enable us to understand how we can frame and communicate findings most effectively. We anticipate disseminating findings through multiple routes, including blogs, presentations, summary reports, academic journal articles, infographics, video, and social media posts. Recommendations will be similarly co-produced through collaborative work with multiple project stakeholders.

*Status and timeline* of the *study*

We anticipate the following timelines for the study:

- Workstreams 1 and 5 completed by November 2024
- Workstreams 2, 3 and 4 completed by July 2024
- Workstream 6 completed by February 2025
- Study end date: March 2025

## Discussion

This study aims to provide a well-rounded, robust evaluation to inform the delivery of NHSHCs and similar health screening programmes. As stated in the introduction, NHSHC practice various greatly between and across localities, and various modifications to invitation and delivery processes have been made with the aim of improving NHSHC take-up, experience, delivery, or outcomes. A key strength of the study design is the mixed method approach adopted. The multiple workstreams will allow for a broad yet in-depth understanding of the impact of invitation and delivery modifications on outcomes for health check recipients, and how these modifications are experienced by those delivering, coordinating, supporting, and in receipt of NHSHCs. In addition, the inclusion of a dedicated health economics workstream will enable us to incorporate cost and resource information into our analysis. The importance of CVD as a public health issue and the potential to positively impact the incidence of CVD through the successful implementation of preventative screening, means that this study will provide valuable findings for those who seek to optimise the delivery of NHSHCs and similar public health programmes.

As well as contributing to the growing body of evidence on the potential of text messaging to enhance uptake, this study will provide important new insights into the impact of introducing an online booking option for NHSHCs — an area that remains under-researched. The study will also address a gap in research knowledge about the impact on NHSHC outcomes, of delivery by alternative providers such as not-for-profit organisations or healthy lifestyles specialists. It will therefore provide a novel contribution to the public health literature by exploring the impact on health check outcomes of a holistic health check compared with a standard one.

Another novel aspect of this study is the use of the APEASE criteria as a framework through which to understand the modifications under study and their implementation and effectiveness. To our knowledge, this is the first use of the APEASE criteria for the evaluation of an NHSHC programme. The study’s novelty is further increased by the inclusion of PPI representatives as embedded research team members, offering a public perspective on the evaluation process that spans both the breadth and duration of the study.

In addition to contributing to the research literature on NHSHCs through production of academic journal articles, our approach to knowledge mobilisation aims to maximise the practical utility of findings. To ensure maximum relevance and utility of research findings for those involved in the delivery and commissioning of NHSHCs, recommendations for practice and ‘key messages’ for dissemination to different audiences will be co-produced through collaborative working with multiple project stakeholders, including public contributors from the pilot area. This process will involve in-depth consultations, led by the research team, and stakeholder workshops to co-produce appropriate and feasible recommendations for practice.

Our knowledge mobilisation plans aim to ensure that the findings of this evaluation are impactful for the local authority conducting the pilot and its partners and service users, those commissioning and delivering NHSHCs nationally, and for the wider public health community.
